# Surplus embryo donation: terminology and ethico-legal perspectives

**DOI:** 10.1093/jlb/lsaf009

**Published:** 2025-07-15

**Authors:** Roy Gilbar, Sivan Tamir

**Affiliations:** School of Law, Netanya Academic College, 1 University Street, Netanya, Israel; International Center for Health, Law and Ethics, University of Haifa, Haifa, Israel

**Keywords:** adoption, donation, non-personhood, surplus embryos, reproductive autonomy

## Abstract

Numerous cryopreserved surplus embryos are being stored in IVF units in Western countries. IVF patients are required to choose and consent to disposition options for their surplus embryos upon starting treatment. We focus on the option of embryo donation to others for reproductive purposes. The terminology used for this practice is inconsistent, as the term embryo ‘donation’ is used interchangeably with embryo ‘adoption’ in different jurisdictions, government programs, and by private initiatives. Our main argument is that the selected terminology bears conceptual and other consequences for public attitudes, in a way which affects the choice of such a surplus embryo-disposition-option. More specifically, we contend that the pairing of ‘embryo-adoption’ is misguided. We identify material, legal, and policy-related points of distinction between the practices of donation and adoption. Then, we discuss the importance of choosing the *right* terminology, given its power to influence the perception of embryo donation/adoption, and analyze conceptual differences between the two terms, finding only ‘donation’ to be fit for purpose. Next, relying on findings from an empirical study, we consider the effect of the *non*-personhood of the embryo on the appropriateness of each term. Subsequently, we distinguish donation from adoption and justify why the former is more appropriate.

## I. INTRODUCTION

Numerous cryopreserved surplus embryos are being stored in in vitro fertilization (IVF) units across the Western world.^1^ This reality is rather typical to technologically advanced, affluent societies. In some cases, it is boosted by a pro-natalist assisted reproduction policy, yielding a fantastic number of surplus cryopreserved embryos in a given jurisdiction.^2^ The estimated number of cryopreserved embryos in developed countries is high. Some approximations suggest millions.^3^ It is estimated that in the entire United States, the number is >620,000.^4^ Another report cites ~500,000 cryopreserved embryos in 2014, with an additional 20,000 embryos added yearly.^5^ Notably, the decentralization of hundreds of IVF clinics across the USA, combined with private healthcare, is challenging for providing accurate estimates for US surplus embryos.^6^ Other countries similarly face high numbers of surplus embryos, but data are scarce and outdated: In Japan, a 2012 study reported an estimated 61,000 cryopreserved embryos nationwide, 15 per cent of which are stored ‘without definite plans for usage’.^7^ In Australia, 71,000 stored cryopreserved embryos were reported.^8^

IVF patients are required to choose and consent to disposition options for their surplus cryopreserved embryos upon starting treatment. These typically include discarding, donation to research,^9^ continued cryopreservation, and donation to another for purposes of childbearing. Other options for surplus embryos include donation for medical training (UK), donation for quality assurance purposes and potentially for stem cell therapies (Australia^10^); and ‘compassionate transfer’.^11^

Embryo donation can be viewed as an instrumental, medico-social solution to infertile individuals/couples who cannot use their own gametes, or requiring gamete (sperm or egg) donation, as well as for same-sex individuals or couples. From a public health perspective, it could also be key to resolving the resource-intensive surplus embryos conundrum faced by IVF units across the world.^12^

Embryo ‘donation’ is also referred to as embryo ‘adoption’ in different jurisdictions, government programs (eg the Embryo Adoption Awareness and Services program in the USA^13^), and by private initiatives, such as the US Snowflakes Embryo Adoption program,^14^ and the US Embryo Solution.^15^ In light of this interchangeable use of terms, our main argument in this article is that the selected terminology bears conceptual and other consequences for public attitudes, in a way which potentially affects opting for such a surplus embryo-disposition-option. More specifically, we contend that the pairing of ‘embryo-adoption’ is simply misguided, as well as misleading,^16^ for reasons elaborated further below.^17^ Notwithstanding this, we will also deal with arguments supporting the view that the term ‘adoption’ should be used throughout our discussion in this article.^18^

In view of our position, our aim is to analyze and shape the distinction between the implications of using the terminologies ‘embryo donation’ and ‘embryo adoption’, trying to provide legal, bioethical, and sociological justifications for our view, to guide policymakers in regulating this area and provide clarity for surplus embryos donors and recipients.

The article will be constructed as follows. First, we provide a short legal and policy background together with an empirical review to illustrate this practice across various jurisdictions, as well as patients’ attitudes toward the disposition option of surplus embryo donation. In this part, we also report on findings from a study we conducted, examining IVF patients’ attitudes in this area. Next, we consider the appropriate terminology to be applied—embryo ‘adoption’ or embryo ‘donation’—and the power and implications of applying each. We then address the *non*-personhood of the human embryo. After that, we delve deeper into a multi-faceted reasoned argument on why embryo ‘donation’ is more appropriate in the context before us. Finally, we conduct a theoretical discussion of the current situation.

## II. LEGAL AND EMPIRICAL BACKGROUND

### II.A. Overview

Embryo donation for reproductive purposes is permitted in the following countries: the USA,^19^ UK,^20^ Australia,^21^ Canada,^22^ France,^23^ Spain, Italy, Holland, Belgium, Japan, India, Greece, Finland, Singapore, Argentina, Colombia, Uruguay, Romania, Portugal, Venezuela, and the Czech Republic.^24^ Embryo donation is unregulated (the law is mute/silent), or illegal, in the following jurisdictions: Israel, Austria, Denmark, Germany,^25^ Italy, Latvia, Norway, Slovenia, Sweden, Switzerland, China, Taiwan, Turkey, and Tunisia.^26^

In jurisdictions where embryo donation is legally permitted, the policy is based on patient consent. This is the situation for example in the USA, where both the Food and Drug Administration^27^ and the law in some states authorize this practice.^28^

The fact that it is lawful in the USA has led the American Society for Reproductive Medicine (ASRM) to issue its guidance on gamete and embryo donation in 2021. The guidance states that the relevant consent form should indicate, among other things, the period of donated embryos’ cryostorage, as well as alternative dispositions, such as discarding the embryos, where the latter have not been selected by potential recipients.^29^

In the UK, consent to use and store (surplus) embryos is required, according to the Human Fertilisation and Embryology Act 2008 (the HFE Act),^30^ conditional upon prior receipt of counseling, and changeable at any time before embryo transfer.^31^ While the HFE Act does not explicitly address the issue of embryo donation, the practice is permitted and regulated by the Human Fertilisation and Embryology Authority (HFEA), which provides guidance to the public on this issue.^32^ It requires, for example, that embryo donors meet certain eligibility criteria: (a) age 18–35 years for egg donors, and 18–45 years for sperm donors, although clinics could accept donors outside this age bracket in exceptional circumstances; and (b) undergoing health checks required from gamete donors.

In Canada, pursuant to the Assisted Human Reproduction Act, joint consent of spouses or common-law partners is required for embryo donation, with the option for one party to unilaterally withdraw their previously given consent to a specific disposition in a timely manner.^33^

Moving on to the empirical perspective, in jurisdictions where embryo donation is legal, the inclination toward embryo donation seems to vary significantly. For instance, the rate of support for this disposition in the USA is 13 per cent, in Australia—15 per cent, in Denmark—29 per cent, in Switzerland—52 per cent, and in Sweden—75 per cent (with 49 per cent of them supporting embryo donation for reproductive purposes, for *single* women).^34^

Similarly, a 2004 study conducted in Denmark among couples whose frozen embryos were destroyed at the end of the storage period examined the attitudes of IVF patients regarding potential donation of their embryos for specific purposes. Twenty-nine per cent of the participants agreed with the option of donating the embryos to infertile couples, compared to higher rates agreeing to donation to infertility research (60 per cent), stem cell research (57 per cent), and stem cell therapy (49 per cent).^35^

Similar attitudes were found in a multi-institutional US survey conducted in 2007, looking into fertility patients’ disposition preferences for cryopreserved embryos. A majority (59 per cent) was very *unlikely* to donate their embryos to others for reproductive purposes, whereas a mere 7 per cent considered this option as very likely (16 per cent—for patients certain they did not want a baby).^36^ Emerging themes guiding patients’ attitudes in this respect were as follows: ‘not wanting someone else to whom I could donate my embryos raising my genetic child’; ‘my concern that infertile couples to whom I could donate embryos are not screened well enough’; and ‘the feeling that I am responsible for the well-being of any child that could result from my embryos’.

In another study on patient attitudes regarding embryo disposition options, conducted in the USA, it was found that only 13 per cent supported the option of embryo donation for reproductive purposes. When probed deeper into their attitudes toward this option, 78 per cent of participants agreed that donation of embryos *is* the way to help a childless couple, while 73 per cent expressed discomfort with the idea of embryo donation.^37^

A more recent study investigated the selection of disposition options with respect to surplus embryos stored in the IVF units in Israel. The study focused on the communication of information about embryo disposition options available to patients by IVF units’ personnel. It found that although not legally available in Israel, 34 per cent of the participants were willing to donate their surplus embryos to childless couples for reproductive purposes.^38^

Some studies examined the factors that influence motivations to donate. A study conducted on conditional embryo donation, in a private IVF clinic in Australia, found that merely 4 per cent indicated they might donate to other couples.^39^ Forty-eight per cent who expressed a positive view about donation held that donors should be able to specify recipient characteristics, such as age, ethnicity and religion, level of education, and where they live. They interestingly suggested that ‘policies that give donors more control over the fate of donated embryos may improve the acceptability of embryo donation and the willingness of couples to participate and may result in a modest to moderate increase in willing donors’, while responsibly adding that such effect needs to be confirmed empirically.^40^

Interestingly, among study participants who indicated that they were unlikely to donate, 15 per cent responded that the ability to specify such attributes is much more likely to influence the probability of donating embryos. Patients’ reluctance to donate their embryos to other infertile couples stemmed from a ‘sense of ownership’ (‘our children’; the embryo as a full sibling to their existing children) and responsibility for the welfare of the future offspring. Other justifications for participants’ reluctance to donate included concerns about potential consanguineous relationships. This resonates with similar themes emerging from our attitudes’ study (see below).

### II.B. Our Empirical Study

Recently, we have conducted a study examining the attitudes of Israeli IVF patients toward embryo disposition options, including the (yet legally unavailable) option of surplus embryo donation.^41^

Our study was conducted at three IVF clinics across Israel, to include participants from various socio-demographic backgrounds. The study was based on a questionnaire specifically designed for it. Two groups were recruited to the study: (i) IVF patients who received treatment at the time of conducting the study (present patients) and (ii) IVF patients who already concluded their treatment at the time of study (former patients). Present patients were contacted either during their visit in the IVF clinic or between scheduled appointments. Former patients were contacted by phone, and those who agreed to participate were sent a link to the questionnaire.

In total, 286 (former and present) patients participated in the study: 205 present patients and 81 former patients.^42^ The majority of participants were women (84 per cent), Jewish (80 per cent), secular/non-religious (51 per cent), married (81 per cent), with no children (62 per cent), or with one child (25 per cent). Twenty per cent of participants were Muslims, which crudely reflects their proportion in Israel’s population. A majority of the participants had a university degree (55 per cent).^43^

We found that although, to date, embryo donation for reproductive purposes is not permitted under Israeli law,^44^ 62 per cent of study participants responded affirmatively when asked if they would give their consent to donate their surplus embryos to others for reproductive purposes, whereas 19 per cent responded negatively, with no significant difference between former and present patients’ attitudes. However, when competing with different embryo disposition options, only 35 per cent responded they would prefer embryo donation to discarding (65 per cent disagreed with this statement). A similar level of support for embryo donation was seen when confronted with a donation to research disposition option. Only 32 per cent stated they prefer donation to others for reproductive purposes, over donation to research.

To learn about the motivations guiding IVF patients in their position on embryo donation, participants were asked an open question about their view. Various themes emerged in their answers. The main reason for refusal to donate embryos to others for reproductive purposes (~39 per cent) was ‘having genetic siblings running around’, namely, fears of incestuous relations. Another closely related reason was that this is forbidden according to religious (Jewish) laws (25 per cent). Twenty-one per cent cited mistrust in others to raise their biological children, and 12.7 per cent cited potentially needing embryos for self-reproduction in the future.

On the other hand, the most dominant reason for *supporting* donation was helping others who are unsuccessful in conceiving a child (‘everyone deserves to be a parent’). Overall, ~41 per cent of the participants who expressed support for embryo donation indicated this reasoning, according to the following division: 15 per cent expressed willingness to help others, but only once achieving healthy embryos for self (reproductive)-use, while 26 per cent indicated their independent desire to help others’ reproductive efforts.

To better understand participants’ motivations for supporting or rejecting donation of surplus embryos to infertile individuals/couples, we also looked at two demographic variables—sexual orientation and marital status. We asked whether embryo donation is an appropriate act where the donees are same-sex couples or singles. Interestingly, the level of support for donation remained quite substantial. When asked about donation to a heterosexual couple, 65 per cent of participants supported this option. When asked about donation to a male couple, the rate of support fell to 49 per cent. Similar findings were seen for female couples (50 per cent). When asked about donating to a single female individual, the rate of support was similar to that of a heterosexual couple (58 per cent). However, when asked about donation to a single male—the rate fell to 47 per cent (putatively due to the need for surrogacy). In this cluster of questions, we saw a substantial difference between former and present patients, where the level of support for embryo donation was 7–10 per cent higher in the latter group. We speculate that the reason for this could be that present patients who at the time of study are struggling to have a child tend to identify more with childless people—regardless of sexual orientation and marital status—than former patients, who may have already had children.

We shall address below the implications of the findings reviewed in this part. Yet, we would like to highlight at this point that, in our view, these findings send a message that transferring surplus embryos from the genetic parent(s) to childless recipients is not considered by the participants of our study as adoption involves the transference of the legal duty to care for a child from one adult to another. Presumably, if that were the case, we would not have seen relatively high rates of participants willing to transfer their surplus embryos to others.

## III. CHOOSING THE RIGHT TERMINOLOGY

When looking at the legal and medical terminology chosen for allowing IVF patients to offer their embryos to other people for their reproductive goals, two alternate terms dominate the area: embryo ‘adoption’ and embryo ‘donation’. Before justifying our position that ‘donation’ of surplus embryos is more fitting than ‘adoption’, we first demonstrate how the use of the two terms is muddled in practice.

### III.A. Confused Interchangeability of ‘Donation’ and ‘Adoption’

We recognize that there are positions in the literature, in legal frameworks of some jurisdictions and in the clinic, identifying similarity between the two practices, or *de facto* treating them similarly, by perceiving embryo donation as early adoption.

We can find this confused, or hybrid, attitude toward surplus embryos intended for donation/adoption to infertile individuals/couples reflected, for instance, in the USA, within ASRM’s 2021 *guidance* discussed above*.*^45^ Some of the guidance’s language and recommendations are similar to those applied in the context of child adoption, while others are unique to a donation setting, rendering the concept of ‘embryo adoption’ inauthentic or unfitting for the situation.

To illustrate this, take for example the guidance’s recommendations regarding informed consent forms that embryo donors must sign to permit the use of their embryos for the reproductive purposes of others.^46^ These reflect a mixed tendency of donation and adoption in the following ways:

- *Relinquishment of all rights of the donor(s) to the embryo(s) and any child(ren) resulting from embryo transfer*—a mixed donation (relinquishing rights over reproductive tissue) and adoption (relinquishing of legal parental rights) orientation, although more closely related with adoption.
- *Recognition of inadvertent loss or damage to the embryo(s)*—implying a proprietary attitude, and hence—donation oriented.
- *Recognition of the right to refuse the transfer to an inappropriate recipient:*

Adoption laws vary widely between jurisdictions.^47^ However, we can cautiously argue that when compared to closed adoption, such recommendation is seemingly more donation oriented, as biological parents typically cannot voice their opinion regarding the appropriateness of *particular* adoptive parent/s.^48^ However, if we look at open adoption settings, the above ASRM 2021 recommendation could be read as more adoption oriented, for it is quite common for biological parents to be involved in the matching of an adoptive parent/family with their child. In jurisdictions where the involvement of the biological parents is legally acceptable, parents are invited to communicate their preferences.^49^ These could be restricted to the general features of their child’s prospective home (eg, a particular religious or cultural upbringing) or relate to a wider set of adoptees’ characteristics.^50^

Furthermore, the recommendation that donors should receive no compensation for the embryos is perhaps more adoption oriented (ie that paying consideration could be akin to child-selling), although sperm and oocyte donors could (and according to ASRM, *should*) be fairly compensated (or at least reimbursed for their expenses) in some jurisdictions.^51^

Admittedly, some of the recommendations are donation oriented, by nature of the embryo’s *non*-personhood. In other words, they allow consideration of alternatives (eg discarding), which do not apply, and *cannot* be applied, to adoption circumstances. Take for example the recommendation mentioning the ‘[p]ossibility that the embryos will not be selected by potential recipients and that practices can then choose an alternative disposition, such as discarding the embryos’.^52^ If any consideration indicates a pure donation-orientation—it is this one. Opting for the nullifying alternative of discarding is patently not optional for an *actual* child in any setting. This, in fact, demonstrates how incomplete and inappropriate an embryo *adoption* scheme is.

Another point of confusing interchangeability is this: although embryo donors typically do not have a say about who will be the donees of their surplus embryos, some countries offer ‘conditional’ donation, meaning that donors *will* be able to specify recipient characteristics. This could, arguably, give a voice to embryo donors, providing a sense of control over the fate of the embryos and, in turn, reduce donor anxiety.^53^ It could also be deemed desirable for more appropriate, targeted ‘matching’ between (potential) children and recipient families. This seems to somewhat resemble open adoption settings, where, as we mentioned above, parents are offered the opportunity to communicate their preferences regarding adoptive parent(s) characteristics.

To sum up, the interchangeable use of the two terms is far from ideal, but can be explained, as we argue further below. We now move to distinguish donation from adoption.

### III.B. Donation versus Adoption—Setting the Two Apart

In another artificial reproductive technique–related context, that of mitochondrial replacement,^54^ Ravitsky and her colleagues have lucidly illustrated how language frames the ethical debate, and the power of selected terminology on public attitudes, as well as on policy outcomes.^55^ They emphasized the responsibility of ethicists for selecting the appropriate terminology and prompted sensitivity and awareness when choosing certain terminology.

As responsible attentive bioethicists, we naturally support such a view. Applied to the context before us, we hold that the *choice* of terminology—stemming from one’s idiosyncratic mindset—influences, or even designs, an implied or explicit message about the action of giving surplus embryos to others for reproductive purposes. We would further argue that there are some conceptual differences between the two terms and what they intuitively denote, with only one of them—‘donation’—being fit for purpose. Our view relies on the following points.

First, adoption has a more emotive connotation, implying that there is a placement to be made for a *child*. Such placement typically hinges on certain criteria to be met, particularly reflecting concerns for the best interest of the *actual* child put up for adoption. However, this does not fit our context in two aspects: (i) in the embryo setting, there is no—and may never come into being—an actual child ‘whose interests need to be recognized’,^56^ merely a cryopreserved embryo, mostly held to be not more than a *potential* person. This will be further developed in the part below, dedicated to the non-personhood argument;^57^ and (ii) as a result of that, it is not *its* (best) interests which are at stake, but rather—at least from a policy perspective—the right to parenthood of infertile couples or individuals (adoptees/donees), who need others’ embryos to procreate. Their right may stand in contrast to the procreative liberty or autonomy of IVF patients who have surplus embryos and are required to make a disposition decision.

Second, flowing from our previous argument—the term adoption may suggest that the embryo has moral standing and similar rights to those of an actual child, thereby potentially fueling the debate between pro-life and pro-choice advocates. For example, Clark, who investigated the legal aspects of the embryo adoption/donation terminology, notes that using the term adoption ‘raises opposition with abortion-rights groups because it encourages people to view the frozen “spare” embryos as equivalent to children’. Thus, such groups would probably prefer to use embryo ‘donation’ or refer to it as a ‘transfer of genetic material’ from one party to another.^58^

Third, although donors’ perception of surplus embryos may not necessarily be emotionally detached—conceptually, the term ‘donation’, *per se*, is perceived as emotionally neutral (not to mention, factually faithful), whereas adoption invokes a painful connotation of taking a child away from her birth parent(s), who cannot care for her.

Fourth, there is substantial difference between adoption and donation, from the perspective of social justice, or the resources required to fulfill one’s aspiration to become a parent. Although both are types of ‘family-building options in which children are not genetically… related to the individuals raising them’^59^—adoption is associated with scarcity of what we may crudely term as ‘progeny resources’, whereas the amount of available *surplus* cryopreserved embryos is overwhelming.^60^

In addition, on its face, adoption serves a *dual* social and personal justice (for both adopters and adoptees). It allows adoptees to grow in a healthier (carefully chosen) family setting, thereby potentially improving their prospects for thriving and alleviating burdens for society, associated with social welfare support, for example. It also offers (childless) adopters the chance of having a family, thereby alleviating the social health problem of infertility (particularly meaningful in pro-natalist societies), and the opportunity of nurturing a child and having some sense of continuity.

Embryo donation, on the other hand, resonates with the prosocial behavior of altruism (a selfless good deed ‘aimed at improving another’s well-being’^61^) and solidarity, uniquely enabling donees—infertile couples or individuals in need of an embryo (rather than simply sperm or ovum donation)—to realize their parenthood aspirations. As it is well documented that altruism, by itself, can be beneficial for altruists—the personal benefits for donors are limited to these reportedly arising from altruism. Namely, donating surplus embryos to infertile couples/individuals could reward donors with satisfaction and promote their subjective well-being,^62^ especially given the magnitude and exceptionality of the altruistic act (a chance to have a child, carry or rear it from the moment it comes into the world) in our case.

Importantly, both practice and terminology of embryo donation also promote (at least in Israel, where cryopreservation is state funded) a public health interest against the current reality of unethical waste of public resources^63^ (required for maintaining a large number of cryopreserved surplus embryos).^64^ Parenthetically, some repro-technology opponents argue that people should adopt children, rather than use public resources for having genetically related offspring. This attitude is problematic for at least two reasons: (i) the scarcity of ‘progeny resources’ cited above and (ii) that the moral act in most cases would be *supporting* poor and underprivileged parents in raising their children, rather than adopting them.^65^ In any case, using the term [embryo] ‘adoption’ fails to capture this latter cost-related public-health-specific perspective.

Fifth, in a child adoption setting, genetic/biological parents legally relinquish their parental rights over the child through a legal procedure, requiring a judicial order terminating such rights and transferring them to another.^66^ In contrast, in the cryopreserved embryo setting, IVF patients have no parental rights over their embryos, and in the legal jurisdictions we reviewed, such judicial order is currently not required. Thus, the legal procedure is quicker, more technical, and straight-forward than that of adoption. Embryo donors quite simply exercise their reproductive autonomy by legally opting for one of the surplus embryo disposition options available to them. This may entail a broad reading of reproductive autonomy/procreative liberty, to include what are in fact *non*-reproductive or *other*-reproductive choices. Namely, while IVF patients decide to renounce the reproductive potential of their (surplus) embryos for themselves, they use their reproductive autonomy (as other proprietary rights would seem inappropriate here) for aiding others in achieving *their* reproductive goals.

Sixth, the term ‘donation’ is wider in scope than ‘adoption’ and goes beyond the realm of family building. One could imagine that the proliferation of the term and its application to a wider set of circumstances could be educative for the public, bringing higher engagement with positive prosocial behaviors (ie organ donation, blood donation, money donation, etc.).

To conclude, using the term ‘adoption’,^67^ with its emotionally loaded, life-changing connotation, could somewhat deter IVF patients from making a decision to transfer their surplus embryos to others for the latter’s reproductive purposes. This stands in contrast to the more pragmatic, neutral, and emotionally detached terminology of ‘embryo donation’. As argued above, we believe that the use of a particular terminology influences reality. It also carries normative implications.^68^ Thus, in our view, the ‘wrong’ terminology could hinder policy implementation, while the ‘right’ terminology could advance a solution for the surplus embryos conundrum.

To illustrate this, using the term ‘embryo adoption’ could compel policymakers to apply adoption-similar scrutiny measures, such as more stringent eligibility criteria for embryo ‘adopters’, for screening recipients and for optimally pairing between embryo donors and recipients through attempted matchings.^69^ It could also entail harsher scrutiny of the (now-embryo) future child’s placement, similar to conducting a *Child Protection Order Check.*^70^ Pragmatically, this could necessitate greater resources from social services, as well as from IVF units. Given the mere potentiality of such ‘future children’, such expenditure could be deemed an irresponsible waste of public funds. Furthermore, it could unjustifiably burden embryo donors and recipients, emotionally and otherwise, potentially hampering ‘putting surplus embryos up for adoption’. Presumably, all this and more would be counterproductive for resolving said reality of surplus embryos and will be to the detriment of individuals or couples suffering from infertility.

Another key conceptual issue distinguishing donation from adoption with a significant implication for terminology is that of personhood, or rather—the lack of it, as far as cryopreserved embryos are concerned. We turn to discuss it next.

### III.C. The Non-Personhood of the Human Embryo

Determining the moral status of the human embryo in a given jurisdiction is essentially relevant for all embryo disposition options (continued storage in cryopreservation, donation to research, discarding, and donation for reproductive purposes). It is also relevant to therapeutic techniques such as mitochondrial replacement technique. We divide the discussion in this part into bioethical and legal perspectives of embryo non-personhood.

Bioethically, the issue of personhood carries significant weight in the context of embryo donation/adoption. As the review conducted in the previous part shows, it seems to influence IVF patients’ level of attachment to the embryo, and consequently their willingness to depart from it, knowing that another individual/couple may raise their genetically related offspring. Naturally, if an embryo is ascribed personhood, disposition options such as discarding or donating to research (involving embryo destruction), would be deemed morally unacceptable. So, we could confidently determine that embryo personhood would thwart various surplus embryo disposition options, not to mention the problematic consequences of fetal personhood for women’s reproductive autonomy in the context of pregnancy and its termination.^71^

In this context, public attitudes toward the moral status of embryos have been empirically investigated. For instance, a survey conducted across nine US fertility clinics found that 18 percent of patients ascribed full moral status to their embryos—a perception which has informed their surplus embryo disposition decisions.^72^ Furthermore, our study found that over half (55 per cent) of the participants (former and present IVF patients) agreed with the statement that an embryo conceived in the laboratory has the same rights as a human being. The rates increased somewhat for embryos implanted in uterus. Sixty-two per cent of participants considered them to have the same rights as human beings. These findings accord with the rate of participants (64 per cent) who disagreed with the statement that a fetus has rights only from birth. Overall, these findings suggest that people perceive an embryo as an entity that has moral status (ie ascribing personhood to the embryo). This, in turn, may support the view that the transfer of embryos to others for reproductive purposes should be regarded more as adoption than as donation.

However, delving deeper into the personhood/non-personhood discussion—it is apparent that on an imaginary spectrum, the moral perception of the human embryo stretches between potential persons of high moral status (invoking rights and meriting protection)—to a mere reproductive tissue or cluster of cells, non-persons with no moral standing or rights to protect.^73^ In between, there is ample room for a gradualist approach, ascribing higher moral status and rights as the embryo develops [embryo-fetus-child (full person)).^74^ This was reflected in our study, where 68 per cent of the participants agreed with the statement that the rights of the fetus in the womb progressively develop throughout the pregnancy.^75^

The embryo personhood debate in bioethics seamlessly intertwines with our former argument about choosing the right terminology. The close link between the two is articulated in an interview given in 2016 by Owen Davis, then-president of ASRM. Commenting on the effect of terminology on embryo personhood he stated that: ‘[T]erminology is very important…. These frozen embryos could not possibly survive outside the body. Their cells have not differentiated, not become a fetus, and certainly not gestated and delivered.’^76^ In the same news report, Barbara Collura, head of Resolve—the US National Infertility Association, pronounced that ‘[A]ttributing “personhood” to donated embryos has “dangerous” implications for both abortion rights and other forms of fertility treatment’.^77^

ASRM’s 2023 ethics committee opinion on ‘defining embryo donation’^78^ also addresses the legal effects of the terminology applied to the embryo’s moral status, stating that

…the term “adoption” … reinforces a conceptualization and status of the embryo as a fully entitled legal being and may lead to a series of legal procedures required for the adoption of born children.

Clearly, this should not be the case for embryo donation.

These statements, in our view, highlight a pragmatic perspective, which reflects the close link between terminology and policy. Negating embryo personhood could potentially be productive for alleviating the storage burden of cryopreserved embryos in IVF units (in jurisdictions facing such conundrum). It could facilitate the psychological perception of the process for IVF patients, thereby promoting the abovementioned public health interest in avoiding unethical waste of public resources (eg in Israel). It could also save IVF patients with surplus embryos the unwarranted socio-legal constraints and complexities involved in child adoption.^79^

Moving on to examine the legal perspective of (non-)personhood, one finds that the legal position on the moral status of embryo varies between jurisdictions. In the UK, the HFEA regulation of human embryos addresses the embryo’s ‘special status’,^80^ stating that ‘[T]his “special status” reflects the embryo’s irreducibility to its cellular composition and is a reflection that as humans, we are considerably more than a sum of our parts.’^81^ Practically, an embryo has no legal rights and is situated at one end of the embryo–full-person spectrum. This is apparent when reviewing the legal position on the use of embryos for research.^82^ Such practice is only allowed in the first 14 days elapsed from the creation of the embryo and not beyond (the ‘14-day rule’, currently under debate due to advances in embryology and biomedical research^83^). The 14-day developmental stage represents the cut-off point of the appearance of the ‘primitive streak’ (representing the earliest point in time after which the embryo can no longer split to form twins, ie ‘a marker of individuality’); precedes the development of the nervous system, to avoid risk of causing suffering; and the stage at which the embryo does not possess ‘the qualities which ground our respect for persons—such as consciousness and sentience’.^84^ And so, the underlying moral position is that at this 14-day stage the embryo has no status at all, so it can be used for research and then discarded.^85^

Another indication for the position of English law, which ascribes no status to an embryo, is seen when we compare the legal status of the embryo to that of a fetus gradually developed in the womb. Although under English law a fetus has no legal rights, some of its interests are legally recognized.^86^ Yet, distinctively, English law does not acknowledge these interests when it concerns an embryo.^87^ As Mary Warnock pointed out, this legal position reflects the reality that there is no guarantee that an embryo would become a person. As medicine teaches us, the chances are relatively modest.^88^

However, the legal position on embryo (non-)personhood is quite different when we cross the Atlantic. Recently, in the landmark case of *Dobbs v. Jackson Women’s Health Organization*,^89^ the US Supreme Court overturned *Roe v. Wade*^90^ and *Planned Parenthood v. Casey*^91^—the decisions asserting the fundamental constitutional right to an abortion (prior to fetal viability). As the Court explicitly avoided making any assertions about fetal personhood, it was left to states’ legislative bodies.^92^ Some states have consequently hurried to pass laws banning abortion, thus impliedly ascribing personhood to fetuses, while others have not.^93^ Naturally, there is a distinction to be made between fetuses and pre-implanted, cryopreserved (surplus) embryos—our point of concern here. However, it shows that there are laws and policies in place that attribute no legal rights nor moral status to embryos and fetuses, whereas others, who adopt a more conservative and religious view, ascribe personhood to them.

An issue that arises from this bioethical and legal debate is whether personhood ascribed to unborn children, fetuses, and embryos *in utero* extends to *in vitro* embryos. This has been the point of contention in a case brought before the Alabama Supreme Court, in the context of a wrongful death lawsuit for the accidental destruction of cryopreserved embryos. In a controversial ruling issued in February 2024, the Court reversed the trial court’s decision that ‘a frozen embryo is not a “child” under Alabama law’,^94^ and held that ‘unborn children are “children” for purposes of Alabama’s Wrongful Death of a Minor Act’, perceiving embryos created through IVF as ‘extrauterine children’.^95^ The ruling has been heavily criticized^96^ in the media, in the literature, and by IVF clinics and practitioners, even creating a backlash for the latter throughout IVF clinics in Alabama. Several IVF clinics halted treatments after the said ruling, resuming their operations only after the governor signed legislation (SB159),^97^ providing broad criminal and civil immunity to Alabama IVF clinics for embryo death or damage during the IVF process.^98^

To conclude this part, those, like us, who do not ascribe personhood and consequent moral standing to embryos would support applying the term ‘donation’ in the context before us. This leads us to the next part, where we showcase the better appropriateness of the ‘donation’ terminology, for legal and practical reasons.

## IV. WHY EMBRYO ‘DONATION’ IS MORE APPROPRIATE

So far, we have focused on opting for the right terminology for the act of transferring surplus embryos to others for reproductive purposes. In this part, we take a step further and distinguish the two practices and the relevant implications of using these terms. The discussion here adds another layer of support to our view that ‘donation’ is the right term to be used.

### IV.A. Distinguishing the Practice of [Surplus Embryo] Donation from [Surplus Embryo] Adoption

In this section, we argue that there are significant differences between the two family-building practices—adoption and donation—which justify keeping to a uniform term of ‘embryo donation’. The following points and reasons support our view.

#### IV.A.1. Legal and policy-related reasons

From a legal perspective, adoption involves the permanent legal transfer of parental rights and duties, and the social responsibility for the child put up for adoption, in a way that ensures or promotes her well-being.^99^ Adoption severs one legal relationship (termination of the biological parent(s) legal rights), while creating another, in a way that affects the personal status of the biological parents, the child candidate for adoption, and the adoptive parents. In contrast, in an embryo donation setting, IVF patients essentially authorize the legal transfer of rights over *reproductive tissue* to others.^100^

Furthermore, in adoption, the state, through its judicial system, has the sole authority to determine whether a child would be placed for adoption. It thus has the authority to terminate the biological parents’ legal responsibility for the child (as legal guardians). This cannot be determined by an agreement between the biological and the adoptive parents.^101^ The state must be involved. Appropriately then, the government as *parens patriae* plays a substantial role in the removal from home and adoption of existing children.^102^ Conversely, the process of transferring surplus embryos from the genetic parents to a couple/individual that need it for procreation can be carried out through the parties’ agreement alone, subject to the regulation in place, with minimal or no government involvement.^103^

In addition, whereas in many jurisdictions, the welfare of the child or his/her best interests are the guiding principle when considering decisions that may affect his/her life,^104^ such as in the case of adoption, there is no such legal guiding principle in place for the ‘best interests’ of the embryo.^105^ In other words, there is no need to consider the future interests and well-being of the child who may or may not be born out of a particular embryo. The obvious conclusion is this: transferring the embryo from the genetic parents to the recipients does not effectively change the legal status of the embryo. In our view, adoption is therefore fundamentally distinct from embryo donation.

This point seems consistent with ASRM’s 2023 ethics committee opinion.^106^ The guidance states that using the term ‘adoption’ in the context of embryo donation is ‘inaccurate and misleading’, and that ‘[e]quating an embryo with a child and applying the procedural requirements of adoption designed to protect children to embryos is *neither ethically nor legally justifiable and has the potential to harm*’ [emphasis added by authors].^107^

It further states that

…embryo donation decisions are finalized and memorialized before an embryo is transferred to a recipient, and no change of mind by an embryo donor following the initiation of a pregnancy would be appropriate, or acceptable, to any embryo donation participants.

In contrast, ‘adoption laws in almost every state require a “cooling off” or change-of-mind period’.^108^

ASRM’s 2023 guidance also importantly highlights another related issue. It makes the point that individuals struggling with infertility and requiring embryo donation equally deserve procreative privacy, as do individuals enjoying natural procreation.^109^ Subjecting the former to the penetrative scrutiny involved in child adoption (but spared to some extent in embryo donation settings)—to ensure the best interests of the ‘embryo adoptee’—is an unwarranted breach of their intimacy and procreative autonomy. We agree with this position.

Finally, another legal, jurisdiction-specific point is that gestation could ‘remedy’ genetic non-relatedness or non-parental ties, thereby replacing the need for an adoption process. For instance, in some jurisdictions, the woman who carries the fetus to term is considered biologically and legally as the mother of the child, and her legally bound partner—as the child’s father.^110^ This legal perception in fact renders adoption and adoption-related terminology redundant for the setting of embryo donation, where the embryo is gestated by the donee.

#### IV.A.2. Asymmetry between the benefiting parties

The identities of the benefiting parties in typical donation and adoption situations are not symmetrical: the benevolent act of adoption—involving reciprocity in doing good, for both adoptees and adoptive parent(s) (a ‘win-win’ situation)—is not the same as the generous act of donation by IVF patients to embryo recipients. Following the non-personhood argument—as opposed to a child adoptee—the embryo, carrying no moral status, cannot be benefited (nor harmed) by being donated to other individuals for reproductive purposes. In other words, it does not have a right to be carried to term and being born. The donors’ benefit seems somewhat limited to the satisfaction attached to altruistically performing a good deed (helping infertile individuals/couples build a family).^111^ In adoption settings, it seems difficult to argue that biological parents are directly benefited by the act of adoption (other than the forced argument of comforting themselves with the knowledge that their child/ren may be raised in a better home). Society, however, is a beneficiary in both settings, albeit with different trajectories: in the donation setting, public health is being promoted (by providing a remedy for infertility), and in the adoption setting—social welfare.

#### IV.A.3. *‘Surplus [embryo] adoptees’?*^112^

Another argument is that when considering language in the context before us, one cannot avoid the unease in linking ‘surplus’ to adoption, as morally—there could never be ‘surplus children’ or ‘surplus human beings’ (as a continuum of the original embryo). In contrast, ‘surplus embryo’ donation (of procreative tissue, a cluster of cells with human potentiality) does not seem to invoke such sentiments of unease.

#### IV.A.4. (In)voluntariness, parenthood, and distinct journeys

Both donation and adoption involve the severance of bio-genetic ties, but evidently embryo donors’ patient journey is quite distinct from biological parents’ parenthood journey (in adoption settings). Whereas donation is more closely associated with a generous, willing act at the end of a long and complex patient journey (to achieve embryos through IVF), adoption is distinctly linked not only with voluntary, but also with *involuntary* (forced) relinquishment of one’s ‘parent-journey’.

#### IV.A.5. Embryo donation’s dual designation

Noteworthily, unlike child adoption, embryo donation carries an anomaly. There is duality in the designation of surplus embryos: on the one hand, there is the option of donating an embryo to research, which is known to result not only in the *non*-realization of its life potential, but in its destruction. On the other hand, there is the option of donating it to another couple/individual, with the very aim of realizing its life potential and bringing a child into the world. For obvious reasons, no such duality exists for child adoption. This duality may support the perception of transfer of surplus embryos as donation, rather than adoption.

#### IV.A.6. Donor/parent anonymity and the right to know one’s genetic origins

The right to know one’s genetic origins, that is—narrative information about the identity and medical and social status of one’s authentic genetic progenitors, is gradually recognized as a fundamental tenet of one’s autonomy and self-determination.^113^ In (non-open) child adoption settings, adoptees could typically access sealed adoption records to find out the identity of their biological parents, or in order to receive pertinent medical information, upon reaching the age of majority (18 or 21). Conversely, in embryo donation settings, the identity of the genetic parents (embryo donors) can only be revealed in countries that legally removed gamete and embryo donor anonymity, out of deference to the right to know one’s genetic origins. And while a growing number of jurisdictions are shifting toward an open-identity paradigm [eg, Sweden (the pioneer, in 1985), UK (in 2005), the Netherlands, Austria, Switzerland, Finland, Iceland, Norway, New Zealand, and some Australian states], others (including most US states and Israel) have opted to retain donor anonymity. This may indicate that the significance attributed to knowing one’s bio-genetic source is more intuitively connected with child adoption than with ‘mere’ embryo donation.

Laying out the implications of using a donation/adoption terminology and inferring that donation is the more appropriate term do not conclude the debate. Notably, some scholars argue that the term ‘embryo adoption’ is in fact more adequate. We will critically deal with this view next.

### IV.B. Grappling with Embryo ‘Adoption’ Enthusiasts^114^

Supporters of the ‘embryo adoption’ terminology rely on two perspectives: one is empirical and the other is bioethical/philosophical.

Empirically, proponents of the perception of embryo adoption rely on studies that reflect the difficulties of donors to donate ‘something of the two of us’, or in other words ‘a symbol of the relationship’ of the couple, and as something that goes ‘beyond the pure genetic connection’ to the embryo.^115^ Unsurprisingly, it was found that those who hold the above view tend to attach a higher moral status to the embryo than those not holding that view.^116^

We do not deny the validity of this position. However, in our view, it does not justify perceiving the act of transferring the surplus embryos to others as an act of adoption. Our principal approach, as elaborated above, is that a surplus embryo is not a human being. Moreover, the difficulty of giving up or giving away something that is ‘a symbol of close relationship’, something we value dear and has a sentimental value for us, does not necessarily mean that we give this ‘something’ up for adoption. Adoption is defined as the transference of moral and legal responsibility to care for a child from one individual (or a couple) to another. It creates, morally and legally, a parent–child relationship. In contrast, as much as we attribute interests to an embryo, and as much as we may perceive it as a potential human being, its transference to others for procreation purposes simply does not create such socio-legal relationship.

Another empirically based argument concerns evidence indicating that donors find it emotionally difficult to donate surplus embryos to others for reproductive purposes, and that it is their least favorable disposition option.^117^ As much as we can identify with such emotional difficulty, it does not lead, in our view, to the inevitable conclusion that these people give their embryos up for adoption.

Furthermore, we acknowledge that there are studies which show that some donors perceive their embryos as their ‘future children’,^118^ leading them to perceive the act of transferring surplus embryos to others more as adoption than as donation.^119^ This, in our view, does not weaken the validity of our position. It merely emphasizes the link between one’s approach to the moral status of an embryo and the issue at hand—transferring embryos to others for family building. While this link may be obvious to participants in these studies, it does not necessarily impose a duty on policymakers to reflect it. As it is famously known, the law does not necessarily accord with one’s personal views, particularly on normative issues relating to the beginning and end of life.

Next, there are those who justify the terminology of embryo adoption from the bioethical/philosophical perspective. Oliver Hallich argues that ‘[d]onation normally presupposes ownership’, and that since ‘[c]hildren are not our property’—‘we cannot donate them…’.^120^ Hallich then puts forward his main argument. In his view, donors have parental responsibility and owe parental obligations toward their embryos. This view is based on the notion of geneticism, which in short means that those whose gametes created a human being ‘incur parental responsibility.’^121^

One cannot help but think that Hallich presupposes that embryos are synonymous with children (or even with a fetus in the womb), a notion that we disagree with. Whereas some believe that once a sperm fertilized an egg—a human entity is created, and that entity deserves the same protection as a full human being, we do not subscribe to this view. Hence, we cannot agree with Hallich who grounds his position primarily on the concept of parental responsibility, which we deem as overreaching in the case of embryos, who are merely *potential persons*.^122^ Hallich’s position, we believe, could, at best, be exhausted by choosing the characteristics of embryo donees for the sake of the potential child’s best interests, but it cannot lead to the conclusion that the donors give up their surplus embryos for adoption.

We also struggle to accept Hallich’s position because, in our view, it is also irreconcilable with surplus embryo disposition options—legally and practically available in many jurisdictions—of discarding embryos or donating them to research (thereby effectively destroying them).^123^ (It is similarly at odds with the reproductive choice of abortion.^124^)

Hallich also argues that ‘[h]aving responsibilities toward these embryos, whatever it may mean concretely, implies that… [t]he relationship we have toward them is one of parenthood, not one of ownership. Therefore, these embryos cannot be “donated”…’.^125^ Although we might agree with his contention about ownership of embryos (and certainly of fetuses and children), we struggle to accept his principal view that attaches parental obligations toward surplus embryos. Hallich himself concedes that ‘our obligation towards embryos is not to satisfy the needs or interests of the embryo, for… the embryo has no such needs to be satisfied’. Also, his own conclusion that ‘it would certainly be bizarre to equate embryo adoption with the adoption of born children’ is supported by reasons resonating our own.^126^

Finally, examining the normative implications of opting for the embryo adoption terminology, Hallich maintains that ‘embryo transfer is similar to child adoption in that it is a transfer of parental responsibility’.^127^ We argue in response that when embryo donees receive an embryo, if it later becomes a child—*then* they assume parental responsibility. It is not being transferred from the embryo donors, but rather it is the *naissance* of a *de novo* parental responsibility (which did not exist for the genetic progenitors donating their surplus embryos). The notion that ‘parental responsibility towards the embryo does not commit us to the view that we have an obligation to bring the child into existence’ reads like a misnomer, for how can one act as a responsible parent in choosing to destroy one’s embryo (thereby eliminating any chance for a child–parent relationship to ever be formed)?

## V. DISCUSSION

Throughout this article, we argued that the act of transferring surplus embryos to others for reproductive purposes should be construed as donation, rendering the term ‘adoption’ ill-suited. In this part, we analyze the quite ambivalent attitudes of our study participants toward their surplus embryos, comparing them with the attitudes of participants in other studies toward other bodily products. We then continue to compare people’s attitudes toward donating their surplus embryos to others, with people’s willingness to depart from other bodily products. This analysis is important when considering policy in this context.

Significantly, it seems that IVF patients tend to overlook having surplus embryos kept frozen for them in IVF units, generally expressing attitudes of abandonment or neglect toward their surplus embryos, or at least, a lack of interest. This, for example, was manifested in our study, where IVF patients typically failed to actively choose what would be the fate of their surplus embryos.^128^ Generally unaware of the public health costs of such massive cryopreservation and the dire shortage in storing space in IVF units, one may argue that some IVF patients express *indifference* toward the fate of their surplus embryos, particularly once they complete building their family.

This attitude in fact resonates with people’s attitudes toward other bodily products, such as hair, skin cells, blood, and even sperm—naturally or medically separated from them. For example, when undergoing blood tests, people generally express no interest in the fate of their blood sample, *per se*. Herring and Chau provide some other illustrative examples, such as nails, urine, and ear wax.^129^ Thus, arguably, many people knowingly, and *de facto*, *abandon* their bodily products.^130^ In contrast, we could think of other contexts, where people actually present an interest in a bodily product for future purposes, to the extent that they proactively preserve it, as well as willing to pay for its preservation; or where they purposefully designate bodily parts to be donated for the benefit of others.

Let us take for instance the case of umbilical cord blood.^131^ After birth, blood remains in the blood vessels of the placenta and in the umbilical cord that remains attached to it. Umbilical cord blood is rich in hematopoietic stem cells (pluripotent, self-renewing cells, able to develop into all types of blood cells).^132^ Patients who are diagnosed with blood diseases and immune system diseases (such as myeloma, leukemia, and lymphoma)^133^ can be treated by using hematopoietic stem cells, which are typically obtained from bone marrow donation, and in cases where it is not possible to find a suitable bone marrow donor—from umbilical cord blood.

Umbilical cord blood is collected and stored both for donation and for potential future autologous or family use. Umbilical cord blood preservation or donation is proactively initiated by parent(s). Cord blood collection—requiring the newborn mother’s prior consent—is conducted at the end of birth, after cutting the umbilical cord and before the placenta’s exit.^134^ Cord blood preservation is possible both through the public health care system and privately, depending on the regulation of each jurisdiction.^135^^,^^136^ Whereas storing cord blood in public banks incurs no costs, parents seeking umbilical cord blood preservation and opting for the collection, processing, and storage of their child’s umbilical cord blood in private banks pay a fee for such services for the duration of the blood storage.^137^

People all over the world preserve and donate cord blood.^138^^,^^139^ Unlike the case of embryo donation, this phenomenon could serve as an indication to attitudes of solidarity (donation to others), precaution (a ‘“biological insurance” against future disease’^140^), and active involvement people exhibit toward the bodily part of umbilical cord blood (including the willingness to pay the costs it involves).^141^ A 2018 review found, for instance, that ‘[p]arents were found to have positive attitudes toward cord blood donation including *awareness of the value of cord blood and its uses, with the option considered to be an ethical and altruistic choice*’ [emphasis added by authors].^142^

The cord blood example shows that when considering the fate of bodily parts, people *can* take responsibility for their health/bodily management, by proactively making (*self/other*-regarding) decisions, deriving from their right to personal (and in the context before us-procreative) autonomy.^143^

As we have seen, people either demonstrate no interest in their bodily products (thereby, impliedly abandoning them) or they attach particular significance to them, initiating steps for their use/storage and making decisions about their fate. Conversely, one could claim that people’s lack of interest in their bodily products is based on the assumption that they will not further be used by others, and should others wish to use them—particular consent would be sought. In that sense, the attitude toward surplus embryos is similar to that toward other bodily products. Putatively, IVF patients express little interest in their surplus embryos because they trust that they will not be used by others without their consent. However, if this is correct, we remain in the realm of donation rather than that of adoption since, as argued above, adoption—where the welfare of the child is at risk—is not based on consent, but primarily on best interests of the child.

Now, comparing people’s attitudes toward donating their surplus embryos with people’s willingness to depart from other bodily products provides further interesting insight. As the studies reviewed above show, IVF patients express genuine concerns about having their child grow up in another family.^144^ The empirical findings are important, as they imply that IVF patients perceive their surplus embryo as a potential child, rather than as a cluster of cells. Moreover, IVF patients’ relative reluctance to donate embryos seems to be distinctly different from, eg, people’s attitudes toward donating their vital organs (as opposed to renewable bodily products), such as kidneys, or lung or liver lobes. While both share a common aim of creating life or maintaining them, respectively people’s disinclination to donate their organs is associated with the risk posed by donation to their own life (a risky operation, and living life without a vital organ).^145^ In contrast, the reluctance to donate embryos stems from the ‘risk’ of having a genetically related child, a potential sibling to their children, which they would not raise themselves and with whom they would have no contact. This is analogous to the reasons given by biological parents refusing to give up their child for adoption.^146^ Concededly, this attitude is more closely related to adoption than to donation.

This, in our view, may further explain the interchangeable use of the terms donation and adoption, which some find understandable, and we deem undesirable and problematic, as we broadly analyzed above. Alternatively, embryo donation may invoke a hybrid attitude, namely, that the act of transferring surplus embryos to others, for reproductive purposes, involves—at least in the eyes of some IVF patients—both qualities of donation and adoption.

We conclude the discussion with the following argument: people have autonomy over their bodily products. In some cases, or with relation to some products, they take proactive action, expressing solidarity with others (eg sperm/egg donation), whereas with relation to other products they remain passive and (un)knowingly opt for non-solidaric behavior (eg surplus embryos). According to this line of argument, autonomy strengthens the case for ‘embryo donation’ because the decision whether to transfer one’s surplus embryos to others is autonomously that of IVF patients.

## VI. CONCLUSIONS

This article analyzes and discusses the somewhat confused terminology currently used by policymakers, as well as by practitioners, for the act of transferring surplus embryos to others for their reproductive purposes.

Our case for opting for embryo ‘donation’ rather than ‘adoption’ lies on the following arguments: First, conceptually, the term donation intuitively facilitates detachment from surplus embryos and promotes donation for reproductive purposes, as well as for research. Second, using ‘donation’ could serve to avoid confusion with current adoption practices. Third, ‘donation’ is consistent with the position we advocate, negating moral status and human interests for embryos/fetuses. Last, and perhaps most importantly, using the term ‘donation’ could importantly enhance people’s (embryo donors) control over reproductive choice (as opposed to the equivalent, rather limited parental autonomy in the adoption setting)^147^ by allowing them to give their surplus embryos to potential recipients without having gone through the arduous legal and welfare course of adoption.

Furthermore, perceiving the transfer of surplus embryos to others for reproductive purposes as donation leads us to suggest that such solidaric act should be supervised, controlled, and legally regulated by the state. We thus agree with Hallich who argues that ‘we have good reason not to allow genetic parents of IVF embryos to remain passive regarding the future treatment of their embryos they brought into existence’.^148^ Thus, as with similar areas (such as organ donation and surrogacy), donation of surplus embryos cannot be left for private, commercial-based companies or organizations.

The appropriate design of embryo donation–specific policies should involve relevant professionals working in the public health care sector, such as medical clinicians, psychologists/social workers, as well as lawyers and bioethicists. As the findings of our study, as well as those of others, indicate, embryo donation is a highly sensitive issue, raising valid concerns for potential embryo donors (and, possibly, for donees as well). Thus, we believe that to the extent that the law provides the state the authority to regulate and supervise the donation of vital organs, it is all the more so necessary for embryo donation. Establishing such a mechanism would provide the state a substantial level of scrutiny and involvement in the process, as well as protect the interests of all stakeholders, particularly in jurisdictions where embryo donation is privately available.^149^

For those policymakers and bioethicists that are, like us, of the opinion that embryos do not have moral status, and believe that donating one’s surplus embryos to infertile couples/individuals is an altruistic act that should be supported through regulation, or on a policy level—it would be more consistent to use the term ‘embryo donation’ rather than ‘embryo adoption’. This holds for at least two pragmatic reasons: First, the term ‘embryo donation’ would make it easier for IVF patients to consider donation and embark on the process because, as we have mentioned above, it does not invoke the value-laden notion of relinquishing their genetically related potential child. Second, the use of the term ‘embryo donation’ adequately mirrors the actual legal procedure, which is apparently much less demanding than that of adoption of a born child. Evidently, supporters of the view that embryos have moral status, and IVF patients who perceive their embryos as their potential unborn children, will find the term ‘embryo adoption’ more fitting.

To conclude, if one would like to resolve the differences between supporters of the ‘embryo donation’ terminology and those of ‘embryo adoption’, perhaps a third term, departing from either ‘adoption’ or ‘donation’, is required. Or, in other words, if we do not own our embryos, nor have parental responsibility toward them, we might need to come up with a new term that faithfully reflects the nature of the act of transferring embryos to others, for reproductive purposes.^150^ However, until such middle ground is found, we support the use of ‘donation’.

## Footnotes

1 We use the term ‘surplus embryos’ to denote supernumerary embryos, ie ‘abandoned’, untransferred excess embryos—the product of an IVF process—with potential viability following a successful implantation into the uterus. Notably, this term may have a distinctively different meanings in various jurisdictions. In Switzerland, for example, ‘surplus embryos’ are defined as ‘embryos that are produced in the course of an in vitro fertilisation procedure but cannot be used to establish a pregnancy and therefore have no prospect of survival (Article 2 Letter b StRA)’. Federal Office of Public Health (FOPH). Questions relating to surplus embryos. https://www.bag.admin.ch/bag/en/home/gesetze-und-bewilligungen/gesuche-bewilligungen/gesuche-bewilligungen-stammzellenforschung/faktenblaetter/ueberzaehlige-embryonen.html (accessed Jan. 29, 2024). It is also possible to use a more neutral term. Instead of ‘surplus’, which we use here, others employ the term ‘unused’. See: Eric Blyth et al., *Embryo relinquishment for family building: how should it be conceptualised?* 25(2) International  Journal of  Law, Policy & the  Family 260 (2011).

2 See, for example, the situation in Israel: N. Ahituv, *Out of Sight, Life Frozen in Time*, Haaretz Daily, *April* 8, 2022; N. Ahituv, *Revealed: Israel is Storing 1 Million Frozen Embryos, Some From the ‘80s*, Haaretz Daily, April 7, 2022.

3 J. Pieters and M. van Miltenburg, *Altruistic donation of surplus embryos to known and unknown recipients, the Dutch approach*, 16(3) International  Journal of  Fertility and  Sterility 230 (2022).

4 Charlotte Lozier Institute, *Frozen Embryo and the Disposition Decision*, (July 16, 2016), https://lozierinstitute.org/frozen-embryos-and-the-disposition-decision/ (accessed Jan. 29, 2024).

5 Peter A. Clark, *Ethical Implications of Embryo Adoption*, in Craig Atwood and Sivan Vadakkadath Meethal (eds.), Pluripotent Stem Cell Biology—Advances in Mechanisms, Methods and Models (InTech Open 2014), https://www.intechopen.com/chapters/46640 (accessed Jan. 12, 2024).

6 Mary Pflum, *Nation’s Fertility Clinics Struggle with a Growing Number of Abandoned Embryos*, NBC News, https://www.nbcnews.com/health/features/nation-s-fertility-clinics-struggle-growing-number-abandoned-embryos-n1040806 (accessed Jan. 29, 2024).

7 Shizoku Takahashi et al., *The decision-making process for the fate of frozen embryos by Japanese infertile women: a qualitative study*, 13 BMC Medical  Ethics 9 (2012).

8 M. Simopoulou et al., *Discarding IVF embryos: reporting on global practices*, 36(12) Journal of  Assisted  Reproduction & Genetics 2447 (2019).

9 Throughout this article, unless specifically mentioned otherwise, the term ‘embryo donation’ should be read as referring exclusively to donation for reproductive purposes (rather than donation to research).

10 Upon receipt of specific patient consent and appropriate licensing from the National Health and Medical Research Council’s Embryo Research Licensing Committee. See: Reproductive Technology Council, Embryo storage, https://www.rtc.org.au/embryo-storage-2/ (accessed Jan. 29, 2024).

11 A practice where surplus embryos are futilely returned to the patient’s uterus, pelvis, or vagina—at a time when the chance of achieving a pregnancy is negligible—to have the embryos go through a ‘natural’ biological destruction. Sivan Tamir and Roy Gilbar, *Surplus embryos in IVF units in Israel: patients’ attitude towards various dispositions—a bioethical analysis of empirical findings*, 37(1) International  Journal of  Law, Policy and  The  Family*,*  https://doi.org/10.1093/lawfam/ebad020 (2023).

12
*Id*.

13 Office of Population Affairs, *Embryo Adoption Awareness and Services Program*, https://www.opa.hhs.gov/grant-programs/embryo-adoption-awareness (accessed Oct. 30, 2023).

14 Snowflakes, *Embryo Adoption Program, Building Families Together*, https://nightlight.org/snowflakes-embryo-adoption-donation (accessed Jan. 29, 2024).

15 Embryo Solution, https://www.embryosolution.com (accessed Jan. 29, 2024).

16 Others share our view. See Pieters, *supra* note 3.

17 Others use a more neutral terminology, such as ‘relinquishment of unused embryos for family building’. However, since our purpose is to discuss not only the use of the terminology but also its bioethical and legal implications, we use the terms ‘donation’, ‘adoption’, and ‘surplus’. For a more neutral approach, see Blyth, *supra* note 1.

18 For example, Oliver Hallich, *Embryo donation or embryo adoption? Conceptual and normative issues*, 33 Bioethics 653 (2019).

19 See Clark, *supra* note 5.

20 Human Fertilisation and Embryology Authority, *Donating Your Embryos*, https://www.hfea.gov.uk/donation/donors/donating-your-embryos/ (accessed Jan. 29, 2024).

21 IVF Australia, *Embryo Donation*, www.ivf.com.au/sites/ivfa/files/2019-10/cln-001-embryo-donation-26may15_0.pdf (accessed Jan. 29, 2024).

22 Embryo Donation Law, https://www.familyhealthlaw.ca/services/embryo-donation-law (accessed Jan. 29, 2024).

23 K. Bettahar-Lebugle et al., *Embryo donation in France: practice and difficulties. Strasbourg’s experience* 35(2) Gynecol  Obstet  Fertil 114 (2007).

24
https://europeivf.com/en (accessed Jan. 29, 2024).

25 The situation in Germany is in fact somewhat complex. In 2016, the German Ethics Council recommended that surplus embryos’ donation and adoption will be legally regulated, but to date, attempts to regulate it have not been successful. See: https://www.leopoldina.org/en/topics/reproductive-medicine/egg-donation-embryo-donation-surrogacy/; https://www.ethikrat.org/en/press/press-releases/german-ethics-council-recommends-legal-regulation-of-the-donation-and-adoption-of-surplus-embryos/; https://www.drze.de/en/research-publications/in-focus/research-with-human-embryonic-stem-cells/modules/embryo-donation?set_language=en.

26 See Clark, *supra* note 5.

27 The United States Food and Drug Administration. 21 CFR parts 210, 211, 820,1271. *Eligibility Determination for Donors of Human Cells, Tissues, and Cellular and Tissue-Based Products: Final Rule and Notice*. Available at https://www.fda.gov/regulatory-information/search-fda-guidance-documents/eligibility-determination-donors-human-cells-tissues-and-cellular-and-tissue-based-products.

28 See for example, Illinois’ Parentage Act of 2015, https://www.ilga.gov/legislation/ilcs/ilcs4.asp?DocName=075000460HArt%2E+7&ActID=3638&ChapterID=59&SeqStart=6600000&SeqEnd=6799853 (accessed Jul. 17, 2024). However, this legal position may be different or changed in the post *Roe v. Wade* era, in anti-abortion states. That is, pro-life states may limit surplus embryos’ disposition options, eg by sanctioning cross-border embryo transportation for discarding, donating, or performing a compassionate transfer. See: Zachary Walker et al., *Viewpoints on Embryo Cryopreservation and Disposition after the Overturn of Roe V. Wade – An Amazon Mechanical Turk Based Survey, Fertility and Sterility*, Consider This (6 September 2023); https://www.fertstert.org/news-do/viewpoints-embryo-cryopreservation-and-disposition-after-overturn-roe-v-wade-amazon (accessed Jan. 16, 2024). See the position in Louisiana where the law categorizes an ‘in vitro fertilized human ovum’ as ‘a juridical person’ and requires excess embryos be made available, solely to heterosexual, married couples, for ‘adoptive implantation.’ La. Stat. Ann. x 9:129 (1986).

29 ASRM, *Guidance regarding gamete and embryo donation*, 115(6) Fertility and  Sterility 1395 (2021).

30 Human Fertilisation and Embryology Act 2008, 2008 c. 22, §13 and Schedule, www.legislation.gov.uk/ukpga/2008/22/contents.

31 Human Fertilisation and Embryology Authority, *supra* note 18, Schedule 3.

32
*Id*.

33 Assisted Human Reproduction Act 2004, §8.

34 Kjell Wånggren et al., *Attitudes towards embryo donation in Swedish women and men of reproductive age*, 118(3) Upsala  Journal of  Medical  Sciences 187 (2013).

35 S. Bangsbøll et al., *Patients’ attitudes towards donation of surplus cryopreserved embryos for treatment or research*, 19(10) Human  Reproduction 2415 (2004).

36 Anne D. Lyerly et al., *Fertility patients’ views about frozen embryo disposition: results of a multi-institutional U.S. survey*, 93(2) Fertility & Sterility 499 (2010).

37 Alison E. Zimon et al., *Embryo donation: survey of in-vitro fertilization (IVF) patients and randomized trial of complimentary counseling*, 14(8) PLoS One e0221149 (2019).

38 Aviad Raz et al., *Unmet communication needs and moral work in the disposition decision concerning surplus frozen embryos: the perspectives of IVF users*, 274 Social  Science & Medicine. http://dx.doi.org/10.1016/j.socscimed.2021.113804 (2021).

39 Cathline McMahon and Douglas Saunders, *Attitudes of couples with stored frozen embryos toward conditional embryo donation*, 91(1) Fertility & Sterility 140 (2009); M. Simopoulou and others, *Discarding IVF embryos: reporting on global practices*, 36(12) Journal of  Assisted  Reproduction & Genetics 2447 (2019).

40
*Id*.

41 Reference is left out to ensure authors’ anonymity for the blind-review process.

42 Our literature review found that very few such studies were performed on significantly larger cohorts.

43 For a comprehensive detailed account of our study findings, *see* XX [Reference is left out to ensure author anonymity for the blind review process].

44 A 2018 Embryo Donation Bill, creating a non-remunerated, consent-based model for embryo donation, intended to mitigate the surplus embryos problem in Israel and aid infertile (single) women, failed to make it past the preliminary reading in the plenum. See, Embryo Donation Bill—2018, https://main.knesset.gov.il/activity/legislation/laws/pages/lawbill.aspx?t=lawsuggestionssearch&lawitemid=2024574 [in Hebrew] (accessed Jul. 24, 2023). Recently, the Israeli Ministry of Health (MoH) issued guidelines aimed to deal with the enormous number of surplus embryos cryopreserved in IVF clinics. The MoH requires the heads of the IVF units to take proactive steps vis-à-vis the patients, following the initial 5-year storage period, to find out whether they are interested in keeping their embryos in cryopreservation for a fee, for an additional 5 years. Ultimately, the MoH authorizes the heads of the IVF units to discard the embryos if no answer is received from the patients or if they cannot be contacted after several attempts (using email and postal correspondence). See: Ministry of Health, Circular 12/2024 Guidelines for IVF units regarding the dispositions for frozen eggs (Ministry of Health, 2.12.2024), https://www.gov.il/BlobFolder/policy/mr12-2024/he/files_circulars_mr_mr12-2024.pdf (accessed Dec. 9, 2024).

45 See ASRM’s Guidance 2021, *supra* note 29.

46
*Id*. at 1400.

47 See, for example, the differences between the legal position in the USA, China and Bulgaria: Lisa Burden and John Mascolo, *International Adoption Law* (March 18, 2024), https://www.findlaw.com/family/adoption/international-adoption-basics.html (accessed Jul. 11, 2024).

48 See eg the Israeli Adoption Act 1981, §13. See also the English Adoption and Children Act 2002, §47 & 52.

49 See, for example, the legal positions in the UK and the USA: Sensitive Matters, *Adoption in the UK vs the US* (March 1, 2022), https://www.sensitivematters.net/blog/103/adoption-in-the-uk-vs-the-us.

50 Adoption Advocates, *How to Choose an Adoptive Family for Your Baby* (June 21, 2021), https://www.adoptionadvocates.net/how-to-choose-adoptive-parents-for-your-baby/ (accessed Jan. 29, 2024 (US); Adopt South West, *Guide to Adoption*, https://www.adoptsouthwest.org.uk/birth-families/process/ (accessed Jan. 29, 2024) (UK); Canada Adopts! *Choosing Adoptive Parents for Your Baby*, https://www.canadaadopts.com/unplanned-pregnancy/choosing-adoptive-parents-baby/ (accessed Jan. 29, 2024) (Canada); Thinking About Adoption, NSW Government (April 2017), https://facs-web.squiz.cloud/__data/assets/pdf_file/0009/319617/Thinking-about-Adoption-factsheet.pdf (accessed Jan. 29, 2024).

51 See ASRM’s guidance 2021, *supra* note 29.

52
*Id*. at 1400.

53 If it is recognized that embryo donors deserve to have a voice in donees’ selection, why should oocyte or sperm donation not carry the same scrutiny, motivated by the best placement for the future potential donor-conceived child? Policy-wise, this could be highly detrimental to the artificial reproductive technology process (eg making overly specific requirements for donees’ identity, or carrying bias toward certain donees, thereby narrowing the pool of potential recipients). In making this point, we are well aware of the longstanding moral distinction between sperm/oocyte and a ‘complete’ embryo. Nevertheless, such an argument ought to be considered as a possible detrimental implication of applying an ‘embryo adoption’ terminology, with all that it entails.

54
Anne Claiborne, Rebecca English, Jeffrey Kahn J (eds), Mitochondrial Replacement Techniques: Ethical, Social, and Policy Considerations (National Academies Press 2016). https://www.ncbi.nlm.nih.gov/books/NBK355458/.

55 Vardit Ravitsky et al., *The ‘three-parent baby’: a case study of how language frames the ethical debate regarding an emerging technology*, 15 The  American  Journal of  Bioethics 57 (2015).

56 S. Goedeke, K. Daniels, *We wanted to choose us: how embryo donors choose recipients for their surplus embryos*, 36(2) Journal of  Reproductive and  Infant  Psychology 132 (2018).

57 David Heyd, *Embryonic Injuries: Can you sue if you wouldn’t have been born, or born different?* 96(1) Chicago-Kent  Law  Review 145 (200). Notably, Heyd refers to a fetus as a *potential* person, therefore, not qualifying as a person to have the moral interests or rights of persons. Arguably, the case for negating embryos’ moral interests and rights—would be even more compelling.

58 See Clark, *supra* note 5.

59 ASRM, *Defining embryo donation: an Ethics Committee opinion*, 119(6) Fertility & Sterility 944 (2023).

60 See the Introduction section above, and particularly the situation in Israel, *supra* note 3.

61 S.A. Rhoads and A.A. Marsh, *Doing Good and Feeling Good: Relationships Between Altruism and Well-being for Altruists, Beneficiaries, and Observers*, in World Happiness Report (2023, 11th ed., Sustainable Development Solutions Network), ch. 4.

62
*Id*.

63 In Israel, where it is estimated that ~1 million surplus embryos are stored in IVF units, the costs of cryopreserving them are high, according to a recent report published by the State Comptroller. Though not providing numbers, the report states: ‘[t]he accumulation of eggs and embryos has a substantial impact regarding… the risks involved in the storage of these eggs and embryos and regarding the cost involved in preserving them… There is a need to renovate some of the IVF units to create more space to store the containers; the units are required to buy more containers, at high cost, and keep maintaining these large amounts of containers, which incurs more costs’. See: The State Comptroller, Special Report: IVF services in Israel – Regulation and Inspection (November 2004) [in Hebrew]; https://www.mevaker.gov.il/sites/DigitalLibrary/Documents/2024/IVF/2024-IVF-Full-Report.pdf (accessed Dec. 10, 2024), 22.

64 See Tamir and Gilbar, *supra* note 11.

65 The authors thank the anonymous reviewer for this insightful comment.

66 Creating a Family, Placed for vs. Given up for adoption, https://creatingafamily.org/adoption-category/placed-vs-given-adoption/#:~:text=adopting%20a%20child%20with%20special,%2Fsurrendered%20her%20parental%20rights%E2%80%9D (accessed Jan. 29, 2024).

67 Notably, adoption does not carry the same meaning all over the world. It is a culturally nuanced, differently regulated practice. For instance, while forbidden in Islam, in the USA adoption of children from different ethnicities, among public figures and celebrities, is somewhat glorified, oftentimes perceived as a status symbol, and as evidence of their cosmopolitanism and anti-racism. The authors thank the anonymous reviewer for this insightful comment.

68 See Hallich, *supra* note 18.

69 Goedeke and Daniels, *supra* note 56.

70 See eg in Victoria (Australia): https://www.mivf.com.au/sites/mivf/files/2019-11/child_protection_order_checks_-_fact_sheet_for_applicants_november2016.pdf.

71 Seema Mohapatra, *An Era of Rights Retractions: Dobbs as a Case in Point*, 48(4) American Bar Association (26 July 2023).

72 See Lyerly, *supra* note 36.

73 We do not discuss here the ‘property-like’ rights of IVF patients with respect to their embryos, as this exceeds the scope of this article.

74 E.H. Huele et al., *The ethics of embryo donation: what are the moral similarities and differences of surplus embryo donation and double gamete donation?* 35(10) Human  Reproduction 2171 (2020).

75 Authors’ published work [reference is excluded to ensure author anonymity for the blind review process]. These findings are also consistent with the findings in the following study: Nitzan Rimon-Zarfaty et al., *When does a fetus become a person? An Israeli viewpoint*, 37 Journal of  Family  Planning & Reproductive  Health  Care 216 (2011).

76 J. Taylor-Coleman, *The Americans Who “Adopt” Other People’s Embryos*, July 18, 2016, https://www.bbc.com/news/magazine-36450328 (accessed Jan. 29, 2024).

77
*Id*.

78
*Id*.

79 See ASRM’s guidance 2023, *supra* note 59.

80 Dave Archard, *Compromises, Fudges and Thresholds*, in Nuffield Council on Bioethics (ed), Human Embryo Culture, Discussions Concerning the Statutory Time Limit for Maintaining Human Embryos in Culture in the Light of Some Recent Scientific Developments (Nuffield Council on Bioethics 2017) 90–93. Abigail Maguire, *An examination into the embryo disposal practices of Human Fertilization and Embryology Authority licenced fertility centers in the United Kingdom*, 30(1) Cambridge  Quarterly of  Healthcare  Ethics 161 (2021).

81 Gill Haddow et al., *Not “human” enough to be human but not “animal” enough to be animal – the case of the HFEA, cybrids and xenotransplantation in the UK*, 29(1) New  Genetics and  Society 3 (2010).

82 Yang Xue and Lijun Shang, *Are we ready for the revision of the 14-day rule? Implications from Chinese legislations guiding human embryo and embryoid research,* 10 Frontiers in  Cell and  Developmental  Biology  10.3389/fcell.2022.1016988 (2022).

83 Insoo Hyun, Amy Wilkerson and Josephine Johnston, “Revisit the 14-day rule”, 533 Nature 169 (2016); John Appleby, Annelien Bredenoord, *Should the 14-day rule for embryo research become the 28-day rule?* 10(9) EMBO Molecular  Medicine e9437 (2018).

84
Emily Jackson, Medical Law: Text, Cases and Materials (6^th^ edn., 2022), 725.

85
Jonathan Herring, Medical Law and Ethics (9^th^ edn., 2022), 500.

86
*Id.* at 402–403.

87
Mary Warnock, Making Babies: Is There a Right to Have Children (2002), 351.

88 NHS, IVF (Oct. 18, 2021), www.nhs.uk/conditions/ivf/.

89
*Dobbs v. Jackson Women’s Health Organization*, 597 U.S. 215 (2022) (No. 19-1392).

90 410 U.S. 113 (1973).

91 505 U.S. 833 (1992).

92 Seema Mohapatra, *An Era of Rights Retractions: Dobbs as a Case in Point*, https://scholar.smu.edu/cgi/viewcontent.cgi?article=2077&context=law_faculty (accessed Jan. 29, 2024).

93 KFF, *Abortion in the United States Dashboard – Status of Abortion Bans in the United States as of September 14, 2023*, https://www.kff.org/womens-health-policy/dashboard/abortion-in-the-u-s-dashboard/ (accessed Oct. 5, 2023).

94 M.R. Laney, *LePage v. Center for Reproductive Medicine, P.C.,*  The Federalist Society (blog) (March 18, 2024) (accessed May 5, 2024); https://fedsoc.org/commentary/fedsoc-blog/lepage-v-center-for-reproductive-medicine-p-c.

95
*LePage v. Center for Reproductive Medicine*, P.C. SC-2022-0515, SC-2022-0579; https://publichealth.jhu.edu/2024/the-alabama-supreme-courts-ruling-on-frozen-embryos (accessed May 5, 2024).

96 Susan Crockin and Francesca Nardi, A*labama Supreme Court Rules Frozen Embryos are “Unborn Children” and Admonishes IVF’s “Wild West” Treatment*, ASRM, https://www.asrm.org/news-and-events/asrm-news/legally-speaking/frozen-embryo-destruction-and--potential-travel-restrictions-for-surrogacy-arrangements2/; David B. Seifer, A *modern-day litmus test for the sustainability of daily IVF practice - Alabama Supreme Court ruling overreaches in asserting that frozen embryos are legally children*, 22 Reprod  Biol  Endocrinol 33 (2024); Sonia Suter and Naomi Cahn, *Lawmakers rushed to protect IVF in Alabama—why wasn’t abortion afforded the same urgency?* 384 BMJ q581 (2024).

97 Alabama Senate Bill 159 – In vitro fertilization; civil and criminal immunity for death or damage to an embryo provided to persons when providing or receiving services related to IVF, https://legiscan.com/AL/bill/SB159/2024.

98 Miranda Nazzaro, *Alabama Governor Signs Legislation Protecting IVF Providers into Law*, The Hill (June 3, 2024), https://thehill.com/policy/healthcare/4514729-alabama-governor-signs-legislation-protecting-ivf-providers/ (accessed May 9, 2024).

99 In an article describing the historical development of adoption law in the USA since 1851, the author states that the primary consideration from the start was to ‘protect children’. See Alice Bussiere, *The development of adoption law*, 1(3) Adoption  Quarterly 3, 5 (1998).

100 See ASRM’s 2023 guidance, *supra* note 59.

101 See eg the Israeli Adoption Act 1981 [in Hebrew] and the English Adoption and Children Act 2002.

102
*Id*.

103 One of us [XX] has written about it elsewhere XX [Reference is left out to ensure author anonymity for the blind review process].

104 See the English Children Act 1989, §1.

105 We appreciate that our view does not correspond with the view of those who support the use of the term ‘adoption’, based on the argument that the couple that created the embryo has parental obligation toward it. See Hallich, *supra* note 17.

106
*Id*.

107 See ASRM’s 2023 guidance, *supra* note 59.

108
*Id*.

109
*Id*.
*Id*.

110 This is, for example, the legal position under Dutch law. See Pieters, *supra* note 3. It is also the case in Israeli law. Under the Embryo Carrying Agreement Act (Agreement Approval and Status of the Newborn) 1996, §11 & 12, intended parents are required to apply to Family Court for a parental order, so that *they*—not the surrogate carrying the child—would be declared and recognized as the newborn’s legal parents.

111 See Pieters, *supra* note 3.

112 A similar, legal terminology-related difficulty is the following: if we opt for ‘embryo adoption’—what do we then call the embryo providers (its genetic progenitors)? It seems non-sensical calling them the ‘genetic *parents*’ as this goes against their intentions of ever becoming parents for any potentially ensuing child. Also, ‘birth/biological parents’ would be factually incorrect. Furthermore, it would be inconsistent with adoption socio-legal terminology for parents whose children have been declared adoptable, or any other definition that implies parental incapacity. So, for this reason too, ‘embryo donation’ (with its factually faithful statuses of ‘donor’ and ‘donee’) seems more suitable.

113 Vardit Ravitsky, *Autonomous choice and the right to know one’s genetic origins*, 44(2) Hastings  Center  Report 36 (2014).

114 For research examining the psychological aspects and implications of donating embryos for reproductive purposes, see the studies of Susan Golombok. See eg Susan Golombok, *Families created by reproductive donation: issues and research*, 7(1) Child  Development  Perspectives, 61 (2013).

115 Veerle Provoost et al., *“Something of the two of us”. the emotionally loaded embryo disposition decision making of patients who view their embryo as a symbol of their relationship*, Journal of  Psychosomatic  Obstetrics & Gynecology, 33(2): 45 (2012).

116
*Id.* at 47.

117 Blyth et al., *supra* note 1, at 267 where they referred to several studies.

118 For example, K. Elford, C. Lawrence, and A. Leader, *Research implications of embryo cryopreservation choices made by patients undergoing in vitro fertilization*, Fertility and  Sterility 81, 1154 (2004); S. de Lacey, *Parent identity and ‘virtual’ children: why patients discard rather than donate unused embryos*, Human  Reproduction 20, 1661 (2005).

119 C. Newton et al., *Embryo donation: attitudes toward donation procedures and factors predicting willingness to donate*, Human  Reproduction 18, 878 (2003).

120 See Hallich, *supra* note 18.

121 See Hallich, *supra* note 18, at 656.

122 See Heyd, *supra* note 57.

123 Although Hallich argues that ‘the thesis of parental responsibility towards the embryo does not commit us to the view that we have an obligation to bring the child into existence’ and therefore such disposition options are among those available to the couple. It also ‘sits well with taking a liberal view on abortion’. We are left unconvinced with respect to both accounts. See Hallich, *supra* note 18, at 657 & 660.

124 A position which Hallich attempts to defend, claiming that it ‘does not mean that the thesis of parental responsibility commits us to a conservative view about abortion. It is neutral with regard to the question of whether abortion is morally legitimate or not.’ See Hallich, *supra* note 18, at 656.

125
*Id*.

126
*Id.* at 658.

127
*Id*.

128 Authors’ published work [reference is excluded to ensure author anonymity for the blind review process].

129 Jonathan Herring and P-L. Chau, *Interconnected, inhabited and insecure: why bodies should not be property*, 40 Journal of  Medical  Ethics 39 (2014).

130
*Id*. at 42.

131 K.K. Ballen et al., *Umbilical cord blood donation: public or private?* 50 Bone  Marrow  Transplantation 1271 (2015).

132 Ji Yoon Lee and Seok-Ho Hong, *Hematopoietic stem cells and their roles in tissue regeneration*, 13(1) International  Journal of  Stem  Cells 1 (2020).

133 Tamila Kindwall-Keller and Karen Ballen, *Umbilical cord blood: the promise and the uncertainty*, 9(1) Stem  Cells  Translational  Medicine 1153 (2020).

134 Ministry of Health, *Conservation of Cord Blood—A Guide for Parents*, https://www.health.gov.il/English/Topics/Pregnancy/CordBlood/Pages/default.aspx (accessed Jan. 29, 2024).

135 See eg the Israeli regulation: The Cord Blood Act 2007 [in Hebrew]; The cord blood regulations (Public Banks) 2012 [in Hebrew]; The cord blood regulations (Private Banks) 2012 [in Hebrew].

136 Apparently, a third storage option exists—that of ‘direct-donation banks’. These are essentially hybrid public and private banks, storing cord blood for both public use, and for families’ own use, for free. https://www.webmd.com/baby/should-you-bank-your-babys-cord-blood (accessed Jan. 15, 2023).

137 Agata Pisula et al., *Women’s attitude towards umbilical cord blood banking in Poland*, 22(4) Cell  Tissue  Bank 587 (2021).

138 Frances Verter, *Percentage of Births Banking Cord Blood by Country* (January 2020), https://parentsguidecordblood.org/en/news/percentage-births-banking-cord-blood-country (accessed Jan. 29, 2024).

139 In Israel, for example, it is reported that several hundred people donate their cord blood to public banks, whereas in 2016 it was reported that several thousand samples are stored in private banks. See: The Weizmann Institute of Science, Davidson Institute, *Cord Blood: Biological Insurance or Gimmick?*  https://davidson.weizmann.ac.il/en/online/reasonabledoubt/cord-blood-biological-insurance-or-gimmick%E2%80%8E (accessed Jan. 13, 2024).

140 American College of Obstetricians and Gynecologists, Committee on Genetics and Committee on Obstetric Practice, *Umbilical Cord Blood Banking, Committee Opinion No. 771*, 133(3) Obstetrics & Gynecology e249, e251 (2019).

141 In fact, such attitude is interesting when it comes to the choice of private banks, given the American College of Obstetricians and Gynecologists’ (perhaps not commonly known) recommendation that: ‘[u]mbilical cord blood collected from a neonate cannot be used to treat a genetic disease or malignancy in that same individual… because stored cord blood contains the same genetic variant or premalignant cells that led to the condition being treated.’ *Id*. at e249.

142 Lisa Peberdy et al., *Parents’ knowledge, awareness and attitudes of cord blood donation and banking options: an integrative review*, 18 BMC Pregnancy  Childbirth 395 (2018).

143 Commenting on English law, Herring addresses the issue of ownership of bodily material: ‘The focus is on consent, rather than property rights…’. Further commenting on whether a living body is considered property, he states: ‘the safest thing that can be said is that there are some respects in which the body can be treated as property, and other respects in which it cannot.’ See Herring, *supra* note 85, at 532, 561.

144 See, the findings from our study, part II, above.

145 L.W. Kranenburg et al., *Psychological barriers for living kidney donation: how to inform the potential donors?* 84(4) Transplantation 965 (2007).

146 John Clifton, *Birth fathers and their adopted children: fighting, withdrawing or connecting*, 36(2) Adoption & Fostering 43 (2012).

147 Interestingly, an empirical study conducted in Iran indicates that donors deem their autonomy highly important in the process of donating their embryos. See: Mahboubeh Taebi et al., *Ethical challenges of embryo donation in embryo donors and recipients*, 23(1) Iranian  Journal of  Nursing and  Midwifery  Research 36 (2018).

148 See Hallich, *supra* note 18, at 660.

149 Our view is supported by the position articulated by the German Ethics Council. See: The German Ethics Council, *Embryo donation, embryo adoption and parental responsibility* (The German Ethics Council, 2019), 121. We thank the anonymous reviewer for directing us to this report.

150 For example, ‘embryo relinquishment’ in Blyth, *supra* note 1.

